# Statistical validation of a global model for the distribution of the ultimate number of citations accrued by papers published in a scientific journal

**DOI:** 10.1002/asi.21335

**Published:** 2010-04-12

**Authors:** Michael J Stringer, Marta Sales-Pardo, Luís A Nunes Amaral

**Affiliations:** 1 Department of Physics and Astronomy, Northwestern UniversityEvanston, IL 60208; 2 Northwestern Institute on Complex Systems (NICO), Northwestern UniversityEvanston, IL 60208; 3 Department of Chemical and Biological Engineering, Northwestern UniversityEvanston, IL 60208; 4 Departament d'Enginyeria Química, Universitat Rovira i VirgiliTarragona, 43007, Spain; 5 Howard Hughes Medical Institute, Northwestern UniversityEvanston, IL 60208

## Abstract

A central issue in evaluative bibliometrics is the characterization of the citation distribution of papers in the scientific literature. Here, we perform a large-scale empirical analysis of journals from every field in Thomson Reuters' Web of Science database. We find that only 30 of the 2,184 journals have citation distributions that are inconsistent with a discrete lognormal distribution at the rejection threshold that controls the false discovery rate at 0.05. We find that large, multidisciplinary journals are over-represented in this set of 30 journals, leading us to conclude that, within a discipline, citation distributions are lognormal. Our results strongly suggest that the discrete lognormal distribution is a globally accurate model for the distribution of “eventual impact” of scientific papers published in single-discipline journal in a single year that is removed sufficiently from the present date.

## Introduction

Citation analysis is a widely-used approach for filtering scientific information. The growth of the R&D workforce, the number of scientific fields, and the number and size of data repositories of research output (Tomlin, 2005) suggest that citation analysis, and other automatic methods of research classification and assessment, are likely to become even more widespread. Despite its increasing usage and importance, there remains deep distrust of citation analysis within the broad scientific community (“Experts still needed”, 2009, “Not-So-Deep Impact”, 2005, Adam, 2002). At the extreme, some critics claim that “the practice is so riddled with errors and biases that it can be worse than useless” (Adam, 2002, p. 729). Thus, it is important to develop methods of citation analysis that reduce errors and biases and that are informed by empirical patterns of citations to scholarly publications (Lane, 2009).

**Figure 1:**
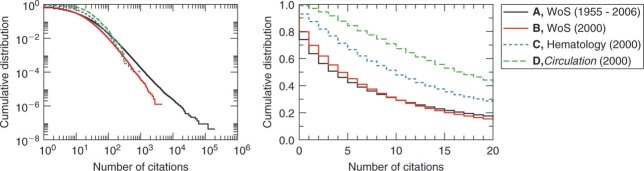
Citation distributions differ by level of aggregation, as well as time period. Citations for all papers in these sets are tabulated at the end of 2006. The left panel is on double logarithmic scales, and the right panel is the same data on linear scale. A, Distribution of the number of citations to all scientific articles indexed in the Web of Science between 1955 and 2006. B, Distribution of citations to all scientific articles published in calendar year 2000. Note that the tail decays faster, for high impact papers have not yet had enough time to accumulate citations. C, Distribution of citations to all scientific articles published in year 2000 in the field of “Hematology.” Note that the median number of citations is significantly (*p* < 0.001) higher in hematology than the median number of citations overall. D, Distribution of citations to all scientific articles published in year 2000 in the journal *Circulation* (which is classified in the field of “Hematology”). The median number of citations to papers published in *Circulation* is significantly higher (*p* < 0.001) than the median number of citations to papers in hematology. At the aggregation level of D, we find that almost all of the data is consistent with a discrete lognormal distribution. Thus, the global distribution A is likely a mixture of discrete lognormal distributions.

The methodological criticisms of citation-based research evaluation typically concern the following three broad aspects of empirically observed patterns of citation to papers:

1. Scientific fields have heterogeneous citation properties, so comparison across fields is unwarranted. For example, computer scientists likely have different publication practices and adhere to different citation norms than sociologists (Wouters, 1999). Although there is much research devoted to defining exactly what a field is, as well as identifying fields using citation data (Shiffrin & Borner, 2004, Boyack, Klavans, & Börner, 2005), the definition and identification of fields remain a difficult problem. An analysis that aims to compare papers, even implicitly in the form of studying a citation distribution, must include only comparable papers to be interpretable in a straightforward manner.
2. Citation counts are dynamic, so evaluations are made before all the information is in. Indeed, the citation count of many papers is still increasing, and almost never assured to remain fixed (Glänzel & Garfield, 2004, Burrell, 2003, Egghe & Rousseau, 2000). Counting the number of citations after a fixed time period biases metrics in favor of fields where paper typically accumulate citations more rapidly. Also, the functional form of the citation distribution depends on the set of years included in the analysis (Simkin & Roychowdhury, 2007). For example, under a simple cumulative advantage model for citation network growth, the global distribution of citations to papers is a power law, but the distribution of citations to papers published within the same year is exponential (Simkin & Roychowdhury; Krapivsky & Redner, 2001).
3. Citation distributions are skewed, so averages are heavily influenced by extreme values. The skewness of bibliometric distributions, especially the skewness in the distribution of the number of citations to papers, has been extensively studied as far back as the 1920's (Lotka, 1926). However, empirical reports of the distribution of the number of citations are often conflicting and depend on the level of aggregation of the analysis (see Figure 1). The models typically used are the power-law distribution (Lotka; Solla Price, 1976; Redner, 1998) and the lognormal distribution (Stewart, 1994; Redner, 2005; Stringer, Sales-Pardo, & Amaral, 2008; Radicchi, Fortunato, & Castellano, 2008), but a number of other skewed distributions have also been considered (Nadarajah & Kotz, 2007). For any of these distributions, the average of a sample is a misleading indicator of the typical value.

Stringer et al. (2008) accounted for these methodological challenges by (a) restricting analysis to papers published in the same journal and year, (b) focusing on the logarithm of the number of citations, and (c) observing the distribution of the number of citations to journal after the papers have stopped being cited. In order to make sure that papers in a set are comparable, Stringer et al. considered separately papers published in the same journal and year, because as one of the primary reasons that journals exists is to group papers by topical relevance (Cole, 2000). An exception to this rule may be large, multidisciplinary journals; they may publish papers that are of interest to a broad readership as opposed to papers that are explicitly related by subject matter. Nevertheless, for a large majority of journals, we expect that papers published within a journal will be comparable in citation properties.

Stringer et al. (2008) showed that within most journals, the distribution of the number of citations to papers within the journal has a characteristic time period, after which the distribution is no longer changing, i.e., the papers in a journal are no longer being cited to an appreciable extent. This suggests that one way to eliminate the confounding issue of citation dynamics is to consider how many citations a paper has accumulated after it has stopped being read and cited. Indeed, the ultimate number of citations that a paper receives may be a more intuitive interpretation of the impact that a paper had on the research community.

Stringer et al. (2008) also showed that a lognormal provides good visual agreement with the citation distribution within journals. Indeed, as shown in Figure 2 of Stringer et al. (2008), the large skewness and kurtosis of the data for all journals lead to the immediate rejection of the hypothesis that the data can be described by classical distributions such as the Gaussian, exponential, or double-exponential.

**Figure 2:**
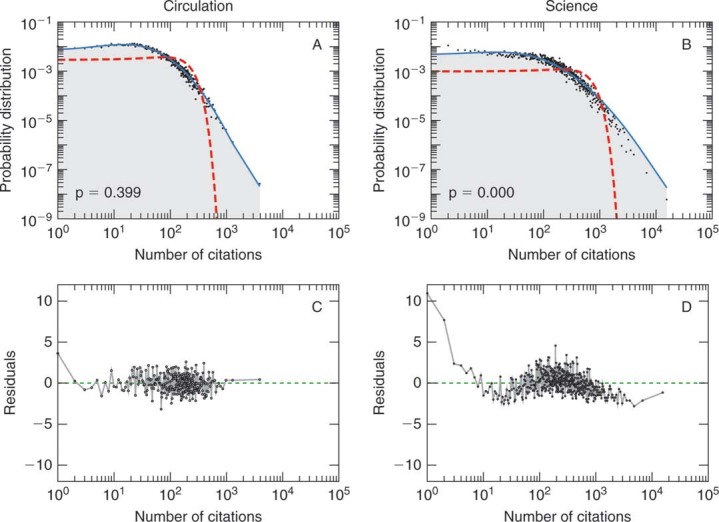
Goodness of fit of latent normal model to journal citation distribution data. A, Comparison of the model to data for articles published in the steady state period (1970–1998) for the journal *Circulation*. We can not reject hypothesis H_1_ (*p*_1_=0.4). B, Plot of residuals, 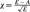 against the independent variable, *n* for the journal *Circulation*. For journals where hypothesis *bf* *H*_1_ cannot be rejected (*p*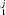 > 0.05), the residuals are uncorrelated with the number of citations. C, Comparison of the model to data for articles published in the steady state period (1996–1998) of the journal *Science*. In this case, *p*_1_ is near zero, indicating that we can reject hypothesis H_1_ with high confidence. D, Plot of residuals, 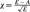 against the independent variable, *n* for the journal *Science*. For journal where hypothesis H_1_ is rejected (*p*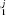 < 0.05), the residuals are correlated. In this particular case, the model under-predicts the number of uncited articles. Whereas for the “true” model of all journal citation distributions, we would expect 5% (109) of the journals to yield *p*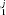 > 0.05, we observe that 10% (229) of the journals yield *p*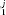 > 0.05. For the purpose of comparison, the dashed lines in A and C indicate best fit normal distributions.

Moreover, there are good a priori reasons to investigate the lognormal distribution as a candidate for the distribution of citations to papers in scientific journals. A lognormal model for the distribution for the number of citations to papers is a plausible model if one assumes that the number of citations that a paper receives depends exponentially on a hidden quantity that aggregates several factors, and that a weakness in any one factor reduces the effect of all the other factors (Stewart, 1994). For example, if a paper must be relevant to current research *and* technically sound *and* visible to the research community *and* clearly written, and so on, then one might expect the hidden variable to be normally distributed and the number of citations to be lognormally distributed (Shockley, 1957).

Here, we use statistical hypothesis testing methods to show that the empirically observed citation distribution is consistent with a discrete lognormal distribution for all but 30 of the 2,184 journals in the Thomson Reuters' Web of Science (WoS) database at an appropriate rejection threshold for multiple tests. For each of the 30 journals that are inconsistent with the discrete lognormal model, we investigate the reasons for the inconsistency. Our findings indicate that for 23 of the 30 journals that fail, the inconsistency is because of the fact that the journal citation distribution is changing “enough” over time to be detected by the statistical test. The seven remaining journals are primarily large, multidisciplinary journals and, thus, likely contain a mixture of papers from different fields with different citation properties. Our results strongly support the hypothesis that a globally accurate model for the distribution of “eventual impact” of scientific papers published in the same journal and year is a discrete lognormal distribution.

## Methods

### Data

We studied citation data from papers in the WoS database, which we gathered using a Web interface available to those with a subscription to the service from Thomson Reuters (http://www.isiwebofknowledge.com). Within the WoS database, we examined papers in the Science Citation Index published during 1955–2006, the Social Science Citation Index published during 1956–2006, and the Arts & Humanities Citation Index published during 1975–2006. All citations counts were enumerated as of December 31, 2006.

To ensure that we do not mix results from different types of published literature, we restrict the analysis to primary literature, which we identify using a series of filters to restrict the papers that we analyze. Before applying any filters, there were 36,658,661 publications assigned to 16,320journals.^1^ We first restrict the papers that we analyze to those classified with a document type of “Article” in the WoS database, which reduces the number of publications to 22,951,535 articles and 16,117 journals. We then further restrict our attention to papers published in journals that contain at least 50 articles per year during, at least, 15 years. This ensures that we can implement the procedure described in Stringer et al. (2008) to identify steady-state periods for the distribution of the number of citations to papers in that journal and analyze the aggregate distribution of citations to papers published during that period. Finally, we consider only journals in which fewer than 75% of the papers remain uncited in the long run—the 68 journals in which more than 75% of papers remain uncited are not primary research literature; they are trade journals such as *Dr. Dobbs Journal*, science news magazines such as *The Scientist*, or non-English language journals that have poor coverage in the WoS database, such as *Measurement Techniques-USSR Journal*.

After filtering, we are left with 12,454,829 primary research articles assigned to 2,184 journals. There is at least one journal included in our analysis from 213 of the 220 fields represented in the 2006 version of the *Journal Citation Reports*. The fields that are not represented are relatively new and do not include any journals that have reached a steady-state distribution. Most of the papers excluded by the filtering process are neither primary literature nor in journals that have been indexed by the WoS long enough to reach a steady-state citation distribution. The remaining excluded publications are in low-volume journals and journals that no longer exist. We find no reason to believe that the low-volume or newly created journals that we exclude from our analysis would exhibit different behavior from the journals that we study.

### Identification of the Steady-State Distribution

The distribution of the number of citations that papers have received changes in time, as papers accumulate citations. To eliminate this confounding effect, we use the heuristic method described in Stringer et al. (2008) to identify periods of time for each journal where the yearly citation distributions are statistically identical, that is, papers in that journal are no longer getting cited enough to change the citation distribution significantly. In cases where we identify multiple steady-state periods in the history of a journal, such as *Ecology*, see Figure 4 of Stringer et al. (2008), we consider only the most recent steady-state period.

### Discrete Lognormal Model

One problem with using a lognormal distribution to model citations to papers is that a lognormal distribution is defined over positive real numbers, whereas citation counts are non-negative integers. There have been a number of ways that researchers have modified the lognormal distribution to account for the fact that citation counts are discrete and include zero counts. Often zero counts are excluded, but this is not appropriate because receiving zero citations is the single most common outcome. Another modification is the delta-lognormal distribution (Aitchison, 1955), which modifies the standard lognormal distribution by allowing a fraction of the papers to have zero citations. In a different approach, Stewart (1994) assumes that citation counts are a result of a Poisson process, with citation rates that are lognormally distributed.

Following Stringer et al. (2008), we make the conversion from a continuous lognormal distribution to a discrete version in the following way. We surmise that the number of citations a paper receives is the result of a latent variable, *q*, and, thus, any paper with a value of *q* in some range will receive *n* citations (Burrell, 2001). In this model, the continuous lognormal distribution would be written as *n*_*LN*_(*q*)=10^*q*^, where we assume that *q* is normally distributed, *q*∼*N*(μ,Ã). Perhaps the “simplest” way of mapping the continuous value of *q* onto the discrete value of *n* is just rounding to the nearest integer. However, because *q* is unobservable, it is not clear what value of *n* should result of a given *q*. Thus, we introduce a parameter g that allows for a discrete mapping that includes the simple floor function (γ=0) and rounding to the nearest integer (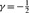). The parameter γ can be interpreted as the value of *q* needed for a paper to get one citation. This assumption leads to the following form for the citation distribution:

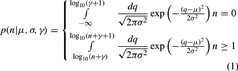


We note that for large values of *q*, the change as a result of discretization is negligible, and the distribution is nearly identical to the lognormal distribution evaluated at only integer values. However, when the mean of the underlying normal distribution is small, the distribution is significantly different from a continuous lognormal distribution. For example, if μ < 0, the distribution *p*(*n*) has a mode at zero and is monotonically decreasing.

### Parameter Estimation Procedure

In the past, one obstacle to using a model where the probability distribution cannot be written in closed form is the prohibitive computational effort required to estimate the parameters of the model. Fortunately, it is now computationally feasible to estimate parameters using maximum likelihood estimation (MLE) in a straightforward manner. MLE is justified in this case because we do not have any prior knowledge about what values the parameters are likely to take for a given journal. To proceed, we, thus, assume that within a journal, *n* is identically and independently distributed. Because the logarithm is a monotonically increasing function, then maximizing the log-likelihood, 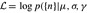, assures we are obtaining the most likely values of the parameters given the observed data. The application of the MLE formalism to this particular case yields:

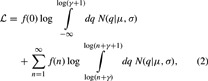

where *f*(*n*) is the number of papers receiving *n* citations. For each journal, we numerically find the parameter values that maximize 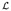 using the downhill simplex algorithm (Press, Teukolsky, Vetterling, & Flannery, 2002).

### Hypothesis Testing Procedures

We test several thousand hypotheses in our study. For such multiple testing situations, one must be careful about the rejection threshold used, as well as the statistical testing methodology (Benjamini & Hochberg, 1995). Unlike a single hypothesis test, where slight nonuniformity in the *p*-value distribution is unlikely to change results, when testing many thousands of hypotheses slight deviations from uniformity in the *p*-value distribution could cause substantial changes in the set of journals that are rejected (Efron, 2007). Furthermore, it is well-known that a large number of false rejections arise when testing multiple independent hypotheses. In this section, we explain each class of hypothesis test that we perform, as well as how we account for the fact that we are testing several thousand hypotheses.

**H**_**1**_**:** The steady-state citation distribution for papers published in a journal is a discrete lognormal distribution.

We use the χ^2^ test to statistically test whether the discrete lognormal model is consistent with the data for each individual journal. The data is binned in such a way that there are at least 5 expected observations per bin based on the maximum likelihood estimate of the parameters. The assumptions necessary for the classical χ^2^ test are satisfied (Taylor, 1997) when it is possible to have five or more bins with an expected count of at least five events, and we assess the significance of the χ^2^ statistic using the χ^2^ distribution with *b*−4 degrees of freedom, where *b* is the number of bins. For most journals, we have on the order of hundreds of degrees of freedom. However, for almost 1% of the journals we consider, we have fewer than 10 degrees of freedom.

Although it may appear to be questionable to fit a three-parameter model to data with fewer than 10 degrees of freedom, we note that we are making the fit to the *same* model for data with hundreds or even thousands of degrees of freedom. Because, as we will see, our three-parameter discrete lognormal model cannot be rejected, it is not appropriate to consider a different model for the small set of journals with only a few degrees of freedom.

Note that when the number of bins is smaller than five, we use a parametric Monte Carlo bootstrap approach with 10,000 bootstrap samples (Efron & Tibshirani, 1994) to assess the significance of the χ^2^ statistic. Such a situation occurs, for example, when the only observed values for the number of citations that papers receive are 0, 1, and 2, allowing for a maximum of three bins.

We denote the *p*-value of hypothesis H_1_ for journal *j* as *p*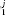. A journal *j* is “rejected” if the observed χ^2^ statistic is unlikely under the null hypothesis, that is, if *p*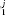 < α_1_, where α_1_ is the *per-comparison rejection threshold*. See the Appendix for a full discussion of the statistical power of this testing procedure.

**H**_**2**_**:** The discrete lognormal distribution describes the citation distribution to every journal.

Another hypothesis we test is whether *all* of the observed data in all 2,184 journals are consistent with the discrete lognormal model. Assuming the citation data is drawn from a discrete lognormal distribution for every journal, the distribution of *p*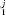 will be uniform between 0 and 1. Using a per-comparison rejection threshold of α_1_ for every test, there is a probability α_1_ that the test will reject the null hypothesis. This process for multiple tests is analogous to flipping a weighted coin some number of times. The actual number of rejections at α_1_ for the set of 2,184 journals is compared to the number expected to occur in a binomial process. Thus, to test whether all of the journals are consistent with the discrete lognormal, we use the number of rejected journals as a test statistic and assess the significance of this statistic, *p*_2_, using the binomial distribution, *B*(2184,α_1_).

**H**_**3**_**:** The steady-state citation distribution for papers published in journal *j* is consistent with the discrete lognormal model, when years are considered separately.

Although H_1_ assumes that the distribution during the “steady-state” period is not changing, we also want to test whether one can reject the hypothesis that the data was drawn from a discrete lognormal distribution, but that the parameters of the distribution vary in time. To test this hypothesis, H_3_, we use the procedure described for H_1_, except we test the hypothesis for each year in the steady-state period separately and the model is rejected for year *y* if *p*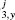 < α_3_. Then, we assess the significance of the number of rejected *years* for journal *j* using the binomial distribution, *B*(*N*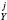, α_3_), where *N*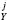 is the number of years in the steady state for journal *j*. We denote the *p*-value of hypothesis H_3_ for journal *j* as *p*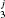.

### Multiple Testing Considerations

Because we are simultaneously testing multiple hypotheses, it is necessary to account for the fact that at a given per-comparison rejection threshold, α_1_, one expects to reject a fraction α_1_ of hypotheses for which the hypothesis is actually true. Keeping the nomenclature consistent with the literature on multiple testing (Benjamini & Hochberg, 1995), we refer to these false rejections as *false discoveries*. For example, if we set α_1_ = 0.05, we expect there to be 2184 × 0.05 ≍ 109 journals rejected, even if the discrete lognormal distributions is the “true” model for every journal.

It is clear that the chosen value of α_1_ governs a tradeoff between the number of false discoveries (drawn from discrete lognormal distribution, but have *p*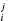 < α^1^) and false negatives (not drawn from discrete lognormal distribution, but have *p*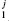 ≧ α_1_). As we are interested not only in how many journals can be rejected but also in identifying common traits of journals that are rejected, it is more important to discover a set of journals, which we can be confident mostly comprises true discoveries. Therefore, using the algorithm described in Benjamini and Hochberg (1995), we set α_1_ = α_*FDR*_ = 0.0007, which controls the false discovery rate (FDR) at a level of 0.05. Therefore, in the set of journals for which *p*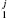 < α_*FDR*_, we expect only a fraction *FDR*=0.05 of the journals to be false discoveries.

## Results

Figure 2 illustrates the goodness of fit of the discrete lognormal distribution for two journals, *Circulation* and *Science*. For 229 of the 2,184 journals in our study, including *Science*, we reject hypothesis H_1_, that the steady-state citation distribution is discrete lognormal, at the α_1_ = 0.05 confidence level. If hypothesis H_1_ is true for every journal in the set, then we expect 109±20 journals to be rejected at α_1_ = 0.05. Clearly, we observe more rejections than would be expected under hypothesis H_2_; thus, we can reject (*p*_2_ < 0.001) that *all* the citation distributions to papers in journals in our study could have been drawn from the discrete lognormal distribution. Note, however, that even for *Science* the fit appears visually to be quite good.

**Figure 3:**
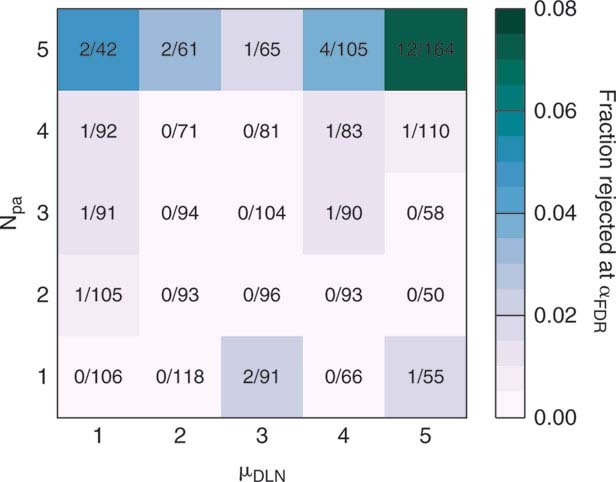
Hypothesis H_1_ tends to be rejected more often for high volume journals. We divide journals in our analysis by quintile according to two attributes: number of papers per annum, *N*_*pa*_, and the best fit mean of the discrete lognormal model, μ_*DLN*_. Most (70%) of the journals for which hypothesis H_1_ is rejected are in the top quintile of *N*_*pa*_. This is to be expected to some extent, since the test has more statistical power to detect even small deviations from the model for larger *N* (see Appendix). However, among the journals in the top quintile of *N*_*pa*_, we find that 57% of the journals for which hypothesis H_1_ is rejected are in the top quintile of μ_*DLN*_. That is, large, highly-cited journals are the most likely to fail the test.

One natural question to ask is whether journals that are *not* consistent with the discrete lognormal model share any traits. For example, if we hypothesize that within a subfield the number of citations is distributed lognormally, then we might expect the distribution to be a mixture of lognormal distributions for journals that span more than one subfield. Figure 3 suggests that the journals for which hypothesis H_1_ is rejected are primarily journals that publish many papers each year. Additionally, among the rejected journals, there is a dependence on μ_*j*_, as there are more rejected journals in the top quintile than would be expected by chance.

The first observation could be at least partially explained by the size dependence of the statistical power of the testing procedure (see Appendix). In journals with more papers, the probability of detecting even small deviations from the discrete lognormal model increases. The second observation, however, cannot be an artifact of the statistical power of the testing procedure, because the power depends weakly on μ_*j*_ (see Appendix). However, it could be that high-impact, high-volume journals are more likely to be multidisciplinary; thus, papers published there would have heterogeneous citation properties.

Under the working hypothesis that papers published within a single subfield will have discrete lognormal citation distributions, the fact that *some* journals fail could suggest two possible explanations: (a) the distribution during the steady state is actually changing, and, thus, the distribution is a mixture of lognormal distributions due to a time-varying mean, or (b) high-volume journals are more likely to publish the research falling within distinct scientific subfields, each having different citation behaviors, and, thus, the distribution is a mixture of lognormal distributions due to heterogeneous citation properties.

**Figure 4:**
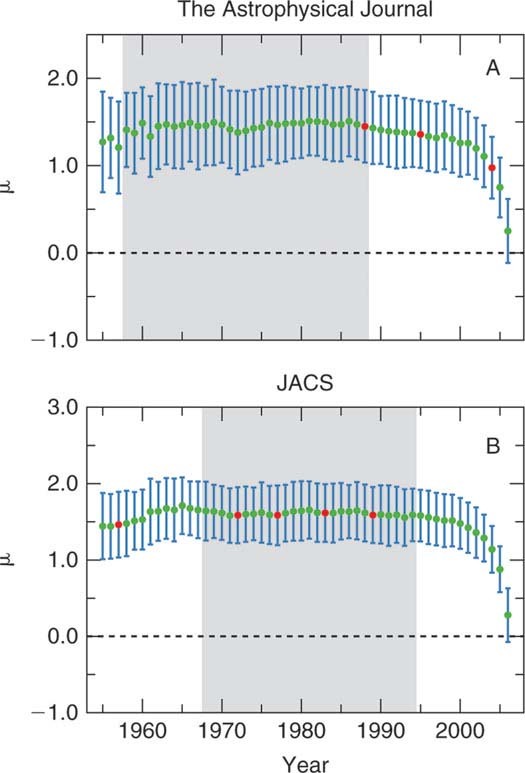
Hypothesis H_3_ is rejected for seven journals. In the set of journals for which hypothesis H_1_ is rejected at α_*FDR*_, some tests fail because the parameters of the discrete lognormal distribution actually vary slightly in time. Panel A shows the mean of the discrete lognormal distribution as a function of time for *The Astrophysical Journal* (Ap. J.). The error bars are intended to show the “width” of the distribution, or the standard deviation σ_*DLN*_, as opposed to the estimated error. For *The Astrophysical Journal*, none of the individual years are inconsistent with the discrete lognormal model, ∀*y*∈{1958, 1959, & 1988}: *p*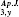 > 0.05. However, when the data from all years in the steady state period (shaded) are aggregated, *p*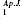 is low enough for hypothesis H_1_ to be rejected with high confidence. In Panel B, we see that for *Journal of the American Chemical Society* (JACS) there are 4 years out of 20 for which *p*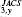 < 0.05. This number of rejections is sufficient to rejected hypothesis H_3_ at *p*_3_ < 0.001. Thus, the time varying mean is not sufficient to explain the deviations from the model expectations for JACS. For purposes of estimating the ultimate number of citations that papers will receive, the heuristic method for determining a “steadystate” is adequate. However, when the number of papers is large enough for the test to be very sensitive, we see that the distribution is actually a mixture of discrete lognormal distributions with a time-varying mean.

The first explanation is plausible because the heuristic method described in Stringer et al. (2008) detects distributions that are statistically similar, but not necessarily statistically indistinguishable. To test if this is indeed occurring, we tested each year in the steady-state period separately using hypothesis testing procedure H_3_. Figure 4 shows the citation distribution history for two journals. Table 1 lists the journals for which the discrete lognormal model fails, using the rejection threshold of α_*FDR*_ (see the Methods section). For 76% of the 30 journals for which the steady-state data is inconsistent with the discrete lognormal model, we found that we cannot reject the discrete lognormal model when each year is considered separately and the multiple testing procedure is used to assess the significance. Thus, it appears that among the rejected journals, the primary reason for rejection is that the mean is changing slightly in time and that the steady state distribution is not really steady “enough.” Table 2 lists the journals for which hypotheses H3 is rejected. Note that multidisciplinary journals are over-represented in this set.

**Table 1:** Journals for which hypothesis H_1_ is rejected, ordered by p_1_.

Journal	*N*_*pa*_	*Y*_*i*_	*Y*_*f*_	*p*_1_	*p*_3_
Journal of the American Chemical Society	1267	1968	1995	0.00000	0.04
Analytical Biochemistry	395	1960	1990	0.00000	0.18
Tetrahedron Letters	1341	1960	1999	0.00000	1.00
Science	921	1988	1996	0.00000	0.00
Physical Review Letters	1860	1970	2007	0.00000	0.01
Journal of Immunological Methods	257	1972	1998	0.00000	1.00
JAMA	536	1996	1998	0.00000	0.00
Journal of Chemical Education	336	1969	2000	0.00000	0.20
Annals of the New York Academy of Sciences	1216	1980	2001	0.00000	0.28
Journal of Molecular Biology	354	1973	1994	0.00000	0.28
Gene	213	1977	1991	0.00000	0.15
Tetrahedron	598	1977	1994	0.00000	0.21
The Astrophysical Journal	766	1958	1989	0.00000	0.80
Journal of Magnetic Resonance	181	1971	1993	0.00000	0.08
JETP Letters	343	1972	2002	0.00000	0.06
Obstetrics and Gynecology	387	1980	1996	0.00000	0.19
American Journal of Physics	175	1961	2000	0.00001	0.59
Archives of Biochemistry and Biophysics	560	1982	1996	0.00002	1.00
Wildlife Research	59	1991	1998	0.00005	1.00
Journal of Organic Chemistry	1023	1969	1990	0.00008	1.00
Arthroscopy	62	1992	1993	0.00028	0.05
Journal of Applied Polymer Science	674	1989	1995	0.00032	0.26
Allergologie	73	1982	2004	0.00034	0.28
Echocardiography	87	1990	2004	0.00041	0.00
Journal of Neurochemistry	332	1958	1998	0.00042	0.28
American Journal of Human Genetics	294	1990	2002	0.00046	1.00
Cereal Chemistry	93	1955	1996	0.00048	0.00
Psychotherapy and Psychosomatics	47	1980	2004	0.00051	0.32
Archives of Dermatology	179	1968	1996	0.00059	0.41
Biochemical Society Transactions	239	1975	2007	0.00060	0.04

*Note*. These journals were identified by setting the false discovery rate to 0.05. That is, we expect up to 4 of these journals to be falsely rejected. *N*_*pa*_ is the average number of papers published per annum during the steady state period. *Y*_*i*_ and *Y*_*f*_ correspond to the start and end of the steady state, respectively. *p*_3_ is the *p*-value for the year-by-year hypothesis testing procedure.

**Table 2:** Journals for which hypothesis *H*_3_ is rejected, ordered by *p*_3_.

Journal	*N*_*pa*_	*Y*_*i*_	*Y*_*f*_	*p*_3_
Science	921	1988	1996	0.00
Cereal Chemistry	93	1955	1996	0.00
JAMA	536	1996	1998	0.00
Echocardiography	87	1990	2004	0.00
Physical Review Letters	1860	1970	2007	0.01
Biochemical Society Transactions	239	1975	2007	0.04
Journal of the American Chemical Society	1267	1968	1995	0.04

*Note*. These journals can be confidently rejected, even when individual years are tested separately. Note that multidisciplinary journals are over-represented in this set. We conjecture that these journals are not consistent with the lognormal model because they are a publication outlet for more than one subdiscipline.

## Discussion

Our results demonstrate that for an overwhelming majority of journals, from every discipline covered by WoS, the distribution of the number of citations to papers published in that journal is consistent with a discrete lognormal model. This implies that for the logarithm of the number of citations, there is a typical value for the number of citations that a paper will ultimately receive, which depends on the journal and year in which the paper is published. For a large majority of journals, this typical value is constant in time after the initial citation accumulation period. The implications of this finding can be valuable for those wanting to compare publications based on their citation impact. Indeed, two of the primary criticisms of citation analysis methodology can be addressed by following two simple procedures: (a) waiting long enough for the journal or paper to accumulate the majority of citations that it will ultimately receive, and (b) using the logarithm of the number of citations as the quantification of impact (as in this case, our intuition about normally-distributed variables holds).

The existence of a normally distributed latent variable and a simple mapping between the value of that variable and number of citations raises the interesting prospect that each journal has a characteristic value for the “citation propensity” or “latent rate” (Burrell, 2003) of articles published therein. This citation propensity could be used to evaluate the effectiveness of journal peer review in selecting papers that will likely have high impact (Bornmann & Daniel, 2008). In addition, in modeling growing citation networks, it could be interesting and useful to explicitly account for the presence of journals and formulate models that reproduce a lognormal distribution of citations. Such models would likely be more realistic representations of the growth of the network of scientific papers.

Interestingly, our results suggest that the global citation distribution over all publications is a mixture of discrete lognormal distributions. In fact, Perline (2005) describes how a mixture of lognormal distributions can mimic a power law, which may explain why many previous studies have reported power law distributions. However, one may equally well ask why the global distribution of citations is important. Scholarly communication practices such as citation behavior, peer review type, typical peer review timescales, and others vary by field (Cole, 2000). Therefore, to avoid obfuscating one's analysis by comparing scholarly publications that are not comparable, it is important to restrict the analysis to fields in which papers have homogeneous citation properties.

## Figures and Tables

**Figure 5:**
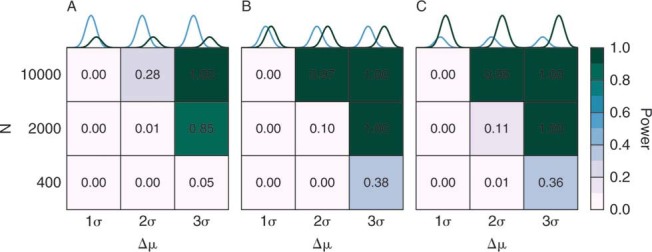
Power analysis of our testing procedure. We draw data from a mixture of two normal distributions with different means but identical variance. 25% (A), 50% (B), and 75% (C) of the points in these samples were drawn from the normal with the largest mean. *N*_sample_ is the number of data points in each sample considered and Δμ is the difference in the means of the distributions. The power of the testing procedure depends on both the specific nature of the deviation from the null hypothesis and the number of points in the dataset. It is clear that a distribution that results from a mixture of lognormal distributions that are separated by only one standard deviation are essentially impossible to detect.

## Footnotes

1 “Journal” is taken as those papers that have the same journal abbreviation in the WoS database. This is the practice that is followed in Journal Citation Reports, and helps aggregate the data correctly.

## Appendix

### Appendix

The probability of having a *p*-value in the rejection region given that the null model is actually false is the power of the statistical hypothesis test. The power of the test depends on the deviation from the null hypothesis, which is unknown for observed data. In addition, the power depends on the number of observed samples. To investigate the power of our testing procedure, we performed a Monte-Carlo simulation experiment of plausible deviations from the null hypothesis. Figure A1 summarizes the results.

We hypothesize that if a journal is multidisciplinary, then it may be the case that the distribution is a mix of discrete lognormal distributions. For simplicity, we consider the case when a journal covers two disciplines with different mean citation propensities and a model in which the latent variable distribution is a mixture of two normal distributions. A fraction *m* of the citation propensities are drawn from the distribution *N*(μ_1_,σ) and the remaining fraction 1−*m* is drawn from the distribution *N*(μ_2_,σ). The means of the component distributions are separated by Δμ. The midpoint between the two component distributions, μ_*C*_ is held constant at a value of 1.0. The value of σ is fixed at a 0.5. The number of samples, *N*_*sample*_ is 400, 2,000, and 10,000. The difference in means, Δμ, is 1σ = 0.5, 2σ = 1.0, and 3σ = 1.5. The fraction of data drawn from the mixture component with the lower mean, *m*, is varied from 0.25, 0.5, and 0.75. For each set of parameters, the following procedure is repeated 10000 times:

1. Draw *N*_*sample*_ numbers from the normal mixture distribution.
2. Estimate the parameters of the discrete lognormal model.
3. Use the χ^2^ as described before to obtain the *p*-value.
4. Reject the discrete lognormal model if *p*≤ α.

Because we know that none of the synthetic datasets are actually generated from the discrete lognormal model, there can be no false negatives, the fraction of times that the discrete lognormal model is rejected is an estimate of the power of the test.
